# Physiological Capabilities of Cryoconite Hole Microorganisms

**DOI:** 10.3389/fmicb.2020.01783

**Published:** 2020-07-31

**Authors:** Ewa A. Poniecka, Elizabeth A. Bagshaw, Henrik Sass, Amelia Segar, Gordon Webster, Christopher Williamson, Alexandre M. Anesio, Martyn Tranter

**Affiliations:** ^1^School of Earth and Ocean Sciences, Cardiff University, Cardiff, United Kingdom; ^2^School of Biosciences, Cardiff University, Cardiff, United Kingdom; ^3^Bristol Glaciology Centre, School of Geographical Sciences, University of Bristol, Bristol, United Kingdom; ^4^Department of Environmental Science, Aarhus University, Roskilde, Denmark

**Keywords:** cryoconite, microbial physiology, cultivation, freeze-thaw, extreme conditions

## Abstract

Cryoconite holes are miniature freshwater aquatic ecosystems that harbor a relatively diverse microbial community. This microbial community can withstand the extreme conditions of the supraglacial environment, including fluctuating temperatures, extreme and varying geochemical conditions and limited nutrients. We analyzed the physiological capabilities of microbial isolates from cryoconite holes from Antarctica, Greenland, and Svalbard in selected environmental conditions: extreme pH, salinity, freeze-thaw and limited carbon sources, to identify their physiological limits. The results suggest that heterotrophic microorganisms in cryoconite holes are well adapted to fast-changing environmental conditions, by surviving multiple freeze-thaw cycles, a wide range of salinity and pH conditions and scavenging a variety of organic substrates. Under oxic and anoxic conditions, the communities grew well in temperatures up to 30°C, although in anoxic conditions the community was more successful at colder temperatures (0.2°C). The most abundant cultivable microorganisms were facultative anaerobic bacteria and yeasts. They grew in salinities up to 10% and in pH ranging from 4 to 10.5 (Antarctica), 2.5 to 10 (Svalbard), and 3 to 10 (Greenland). Their growth was sustained on at least 58 single carbon sources and there was no decrease in viability for some isolates after up to 100 consecutive freeze-thaw cycles. The elevated viability of the anaerobic community in the lowest temperatures indicates they might be key players in winter conditions or in early melt seasons, when the oxygen is potentially depleted due to limited flow of meltwater. Consequently, facultative anaerobic heterotrophs are likely important players in the reactivation of the community after the polar night. This detailed physiological investigation shows that despite inhabiting a freshwater environment, cryoconite microorganisms are able to withstand conditions not typically encountered in freshwater environments (namely high salinities or extreme pH), making them physiologically more similar to arid soil communities. The results also point to a possible resilience of the most abundant microorganisms of cryoconite holes in the face of rapid change regardless of the location.

## Introduction

Ice sheets and glaciers are the biggest freshwater ecosystem on the planet (Edwards et al., 2013), which is undergoing rapid changes (Stocker et al., 2013). It is therefore crucial to understand the biogeochemical processes occurring to predict future changes, their impacts on surrounding environments, and potential losses of functional biodiversity. Complex microbial communities on the surface of glaciers and ice sheets are concentrated in small melt pools called cryoconite holes (Wharton et al., 1985). Cryoconite is a matrix of mineral particles and biological material deposited on glaciers by wind and meltwater, most likely of local origin (Porazinska et al., 2004). Having lower albedo than surrounding ice, it absorbs heat and melts downwards, creating a suitable habitat for microbial life in the supraglacial environment (McIntyre, 1984; Tranter et al., 2004; Cook et al., 2016). The structure of cryoconite holes ensures that the organisms that inhabit them have access to liquid water throughout the ablation season (Fountain et al., 2004; Hodson et al., 2008) and ensures relative high density of different life forms when compared to other supraglacial habitats (Edwards et al., 2011). This also provides protection from extreme fluctuations in air temperature and partial UV screening, either by ice lidding or by the formation of granules (Hodson et al., 2010; Bagshaw et al., 2016): those that form in “cold” ice tend to remain isolated by a refrozen ice lid (Fountain et al., 2004), whereas those formed in regions with more extensive surface meltwater flows experience frequent redistribution (Cook et al., 2010), which promotes the formation of cryoconite granules (Langford et al., 2010). Large granules and thick accumulations of cryoconite material enable formation of anoxic zones (Poniecka et al., 2018), creating a niche for anaerobic microorganisms (Zdanowski et al., 2016).

Rates of microbial activity in cryoconite sediment are similar to those found in temperate freshwater sediments (Anesio and Laybourn-Parry, 2012). Yet microorganisms in cryoconite holes are subjected to multiple stresses resulting from low temperatures and fluctuations of environmental conditions. These include, but are not limited to, freeze-thaw cycles, geochemical extremes including high pH and low nutrient availability, decrease in diffusion rates, increased viscosity of fluids, osmotic stress, and UV exposure. For microorganisms to adapt to this environment, they need to respond to numerous interacting stresses that are usually unspecific (Anesio and Laybourn-Parry, 2012; Collins and Margesin, 2019), and we need to understand how they interact. Cold-environment constraints often induce cross-protection against other stressors. For example, adaption to freeze-thaw stress will also provide protection against heat and cold shock, oxidative stress, metabolic stress (starvation on C or N sources), and/or osmotic stress (Park et al., 1997; Fonseca et al., 2001; Wilson et al., 2012). Identified mechanisms which allow survival of freezing and accompanying stresses include the increased fluidity of the cell membrane (Fonseca et al., 2001; Meneghel et al., 2017), excretion of antifreeze proteins (Park et al., 1997; Raymond, 2016) or other cryoprotectants (Pegg, 2007; Wilson et al., 2012), as well as the production of stress proteins following exposure (Park et al., 1997; Fonseca et al., 2001).

Metagenomic and molecular studies of Alpine cryoconite hole communities have attempted to characterize the mechanisms of adaptation to these extreme stressors. At Rotmoosferner, Austria, it was demonstrated that microbial community members not only have a large array of stress response genes, but that they also have significant genetic potential for effective nutrient and organic carbon scavenging/recycling (Edwards et al., 2013). Utilization of various carbon substrates was also determined in the Austrian Alps (Margesin et al., 2002) and the Himalaya, and also in Antarctica (Foreman et al., 2007; Sanyal et al., 2018). At Forni, Italy, and Baltoro, Pakistani Karakoram, a metagenomic study confirmed the presence of versatile and diverse metabolisms in the cryoconite communities (Franzetti et al., 2016). Genes encoding metabolic pathways of heterotrophic anoxygenic phototrophs and anaerobes were found, as well as enzymes for multiple organic carbon sources such as cellulose, chitin and other polysaccharides [e.g., Extracellular Polymeric Substances (EPS)]. Yet it still remains mostly unknown which groups of microorganisms are capable of effective recycling, if they complement each other or else if they are all efficient scavengers.

Much of the research on polar cryoconite holes has been focused on geochemistry, net ecosystem productivity and carbon cycling (Stibal et al., 2008; Cook et al., 2012; Bagshaw et al., 2013), whilst the actual functionality of these microbial communities remains largely unidentified and physiological limits are untested. Metagenomes of microbial communities on the Greenland Ice Sheet (GrIS) show the potential for resistance to and degradation of anthropogenic contaminants (Hauptmann et al., 2017), but the genetic potential of Antarctic communities has not been investigated. The phenotypic diversity of organisms will affect the robustness of ecosystem and its response to change (Petchey and Gaston, 2006; Srivastava et al., 2019). Ice sheet surfaces are an extreme low temperature environment, but also a very changeable habitat. Cryoconite holes can be saturated with oxygen (Bagshaw et al., 2011) or anoxic (Poniecka et al., 2018); too dark or too light (Perkins et al., 2017); change from hypersaline to low ionic strength (Telling et al., 2014); become acidic or alkaline (Tranter et al., 2004); be frozen and thawed multiple times (Bagshaw et al., 2011); and can be spiked with nutrients or become nutrient limited (Telling et al., 2014; Holland et al., 2019). We therefore hypothesize that the microorganisms that inhabit polar cryoconite holes can tolerate and grow over a wide range of extreme conditions. We further expect them to have a significant potential for scavenging multiple carbon sources. Finally, we predict that Antarctic microorganisms should be able to withstand harsher conditions than their Arctic counterparts. This study presents the microbial ecophysiology of a collection of isolates from cryoconite sediments from sampling locations in Antarctica, Greenland, and Svalbard. The microbial isolates were characterized using cultivation-based techniques in a range of extreme conditions, and the response assessed for each sample location.

## Materials and Methods

### Sampling

Cryoconite material was collected from three polar locations (Antarctica, Greenland, and Svalbard), with three samples from each location analyzed. Antarctic samples (*n* = 3) were collected from ice-lidded cryoconite holes on Canada Glacier in McMurdo Dry Valleys [−77.6175, 162.9734] in the Austral summer of 2005/2006. Svalbard samples (*n* = 3) were collected from Midtre Lovénbreen glacier, approximately 4 km from the United Kingdom Arctic Research Station in Ny-Ålesund [78.8800, 12.0700] in the melt season of 2016. The Greenland study site (*n* = 3), “Black and Bloom” (Williamson et al., 2020), was located 60 km [67.0748, −49.3586] east of Kangerlussuaq, and approximately 2 km east of weather station S6 (Smeets et al., 2018) in the summer melt season of 2016. Antarctic and Svalbard samples were scooped from cryoconite holes using clean, disposable nitrile gloves and transferred into Ziploc plastic bags or tubes pre-washed with deionized water. Ice lids were removed from Antarctic cryoconite holes using a Sipre corer prior to sampling (see Bagshaw et al., 2007). Greenland samples were collected with a pre-washed turkey baster and transferred into sterile Whirlpack^®^ bags. All samples were frozen prior to temperature-controlled transport to Cardiff University, where they were stored in a −20°C freezer until laboratory experiments commenced.

### Total Cell Counts

The total cell count of each of the sediment sample was performed using the epifluorescence microscopy following method of Cragg and Kemp (1995). Briefly, cryoconite sediment from each location (*n* = 9) was diluted 1 to 10 in substrate free medium, then fixed in 1.6% formaldehyde and stained with acridine orange. Total cell counts were determined in three technical replicates with a Zeiss Axioskop microscope after staining.

### MPNs and Cultivability

The Most Probable Number (MPN) technique was used to enumerate viable cell counts in the cryoconite sediment (*n* = 3 for Greenland and Svalbard, *n* = 2 for Antarctica). Following initial dilution of ∼1 g of the sediment in the 9 ml of water, a series of eight 10-fold dilutions in three replicates was prepared on a 96-well plate (Köpke et al., 2005). Samples were grown at temperatures 0.2, 4, 10, 15, 20, and 30°C under oxic or anoxic conditions. For aerobic microorganisms, a freshwater medium was used (Sass et al., 1997), containing the following components: NaCl (0.1 g l^–1^), MgCl_2_ ⋅ 6H_2_O (0.25 g l^–1^), CaCl_2_ ⋅ 2H_2_O (0.1 g l^–1^), KCl (0.1 g l^–1^), NH_4_Cl (0.1 g l^–1^), KH_2_PO_4_ (0.1 g l^–1^), casamino acids (0.25 g l^–1^), and yeast extract (0.05 g l^–1^). The medium was supplemented with 1 ml l^–1^ of the trace element solution SL 10, 0.2 ml l^–1^ of a selenite and tungstate solution (Sass et al., 1997). It was buffered with HEPES (2.38 g l^–1^) and the pH was adjusted to 7.2 with 1 M NaOH prior to autoclaving. After autoclaving, the medium was supplemented with 2 ml l^–1^ of vitamin solution (Wolin et al., 1963) and glucose (4 mM l^–1^). For anaerobic microorganisms, a bicarbonate-buffered fermenter medium was used, containing: MgCl_2_ ⋅ 6H_2_O (0.1 g l^–1^), CaCl_2_ ⋅ 2H_2_O (0.35 g l^–1^), KCl (0.1 g l^–1^), NH_4_Cl (0.1 g l^–1^), KH_2_PO_4_ (0.1 g l^–1^), casamino acids (0.25 g l^–1^), vitamin solution (2 ml l^–1^), glycine betaine (0.5 mM), sodium acetate (0.5 mM), TCA mixture [0.5 mM (Köpke et al., 2005)], choline (0.5 mM), methylamine (0.5 mM), trace element solution SL 10 (1 ml l^–1^), selenite and tungstate solution (0.2 ml l^–1^). The medium was reduced with Na_2_S (1.25 mM) and FeCl_2_ (0.25 mM) solutions. Plates were incubated in air-tight bags with AnaeroGen sachet (Oxoid). MPN values were recorded after 71 days of incubation. Oxic growth was scored after visual inspection of the MPN plates. In anoxic incubations, growth was analyzed after staining with SYBR green I dye (Martens-Habbena and Sass, 2006) and fluorescence analysis on a plate reader. MPN values with standard error and 95% confidence intervals were calculated according to de Man (1983). Viable cell counts obtained with the MPN technique were related to the total counts to estimate culturability.

### Microbial Isolates

The cultivable microorganisms were isolated from the highest positive MPN dilution, therefore representing the most abundant members of community. A sample of 20 μl from the MPN dilution was streaked on to a 1.5% (w/v) agar plate with freshwater medium or anaerobic medium. Anaerobic cultures were prepared in an anoxic chamber and cultured in air-tight bags with AnaeroGen sachet (Oxoid). At least three subsequent subcultures were streaked to obtain a pure culture. Anaerobic cultures were tested for growth in oxic conditions and for alternative electron acceptor utilization (nitrate, thiosulphate, iron, manganese, TMO, DMSO) (Süß et al., 2008).

### 16S rRNA Gene Sequencing

Genomic DNA of each microbial isolate was extracted by bead beating at speed 5.5 m s^–1^ for 30 s (FastPrep 24 Instrument, MP biomedicals) in Guanidine Isothiocyanate lysis buffer (Invitrogen), and then purified with the use of an automated Maxwell 16 Instrument and tissue DNA purification kits (Promega), following the manufacturer’s instructions. Briefly, DNA cleaning steps were performed with the use of magnetic beads binding to the DNA. Genomic DNA concentrations were then quantified using a Qubit 2.0 fluorometer (Invitrogen), following the manufacturer’s instructions.

Extracted DNA was amplified using primers targeting 16S rRNA genes, 27F (5′-AGA GTT TGA TCM TGG CTC AG -3′) and 907R (5′-GGT TAC CTT GTT ACG ACT T -3′) (Webster et al., 2006). Fungal ITS fragment was amplified using primers ITS1f (5′-CTTGGTCATTTAGAGGAAGTAA-3′) (Ihrmark et al., 2012) and ITS4 (5′-TCCTCCGCTTATTGATATGC-3′) (White et al., 1990) in the following PCR conditions: initial denaturation at 95°C for 5 min, followed by 35 cycles of 95°C for 30 s, 56°C for 30 s and 72°C for 30 s; with final extension of 72°C for 7 min. The yield of PCR reaction was visualized on 1.2% agarose gel. Amplicons were then sequenced by Sanger sequencing with 27F or ITS1f at Eurofins Genomics (Germany) or DNA Sequencing and Services (University of Dundee). The nucleotide BLAST online tool^1^ was used to determine the closest relative for each isolate. The 16S rRNA gene sequences determined in this study have been deposited in GenBank under the accession numbers MT430950, MT432272- MT432304, MT473233, and MT473713-MT473721.

### Salinity, Temperature and pH Tolerance

The tolerance of the cryoconite isolates to environmental (temperature) and geochemical (pH and salinity) stresses was tested. Selected isolates were grown in duplicate in liquid freshwater medium at a range of temperatures (1–40°C), salinities (0.1–10%) and pH (2.5–10.5). Salinities from 0.1 to 10% were achieved by adding saturated MgCl (18.75 g l^–1^) and NaCl (290 g l^–1^) solution to the freshwater medium. The pH was adjusted with 1M HCl, with different buffering solutions for pH 5.5–10.5 adopted from Kaksonen et al. (2006). For pH 2.5–5, 100 mM K_2_HPO_4_ was used. Growth was deemed positive or negative by presence of visible cell pellets when compared to negative control (uninoculated freshwater medium) after 30 days of incubation. Differences between microbial isolates’ maximum salinity tolerance according to location, oxic/anoxic isolation or bacterial/yeasts were compared using the Kruskal–Wallis test. Differences in the range of pH tolerated by the isolates were established by comparing the inoculated pH media with positive growth after 30 days. The number of tubes with positive growth at each pH was then compared between locations, oxic vs. anoxic conditions and presence or absence of yeasts using ANOVA, followed by Tukey HSD.

### Freeze-Thaw Survival

To identify whether cells from the isolated polar microorganisms were susceptible to freezing damage, microbial cultures were subject to alternating freeze-thaw cycles in Weiss VT low-temperature environmental cabinets. All of the bacterial isolates from oxic conditions (16) and representative yeast isolates (7 out of 9) were tested. The isolates were washed with mineral medium (NaCl (0.025 g l^–1^), MgCl_2_ ⋅ 6H_2_O (0.09 g l^–1^), CaCl_2_ ⋅ 2H_2_O (0.025 g l^–1^), KCl (0.025 g l^–1^), counted and diluted to equal cell numbers in the mineral medium to exclude the potentially protective effect of substrate-rich medium and to minimize growth in between the cycles (Carvalho et al., 2004).

Each cycle consisted of 6 h at -18°C and 3 h at 0.9°C. Subsamples were taken after 1, 5, 25, and 100 cycles and cell viability was determined using the MPN technique after 30 days of incubation.

### Substrate Test

Substrate tests were set up in 96-well plates as described by Süß et al. (2008) to assess the physiological capabilities of microbial isolates. A total of 58 substrates were tested, including carbohydrates, carboxylic acids, amino acids, alcohols and others. The substrates were chosen to cover a wide range of possible substrates typically produced and utilized by microbes, as well as to cover a range of enzymes needed for different substrates (Supplementary Table 1). Bacterial isolate inocula were washed three times in substrate free medium (freshwater medium with no casamino acids, yeast extract, glucose or vitamins added) prior to the experiment to avoid substrate carry-over. Washed cells were resuspended in substrate-free media and 50 μl added to each well containing 200 μl of medium containing a single substrate. Each substrate was tested at least in duplicate. The wells with positive growth were recorded when compared visually to negative controls (substrate-free medium).

Pearson correlation analysis of the response to the experimental conditions was performed in the “Performance analytics” package in R for each pairwise combination: minimum and maximum pH tolerated, maximum salinity, maximum temperature, average substrate utilization, and freeze-thaw survival for each isolate.

## Results

### Total Cell Counts

Total cells were counted in the sediment samples, which were subsequently used for MPNs. The cell numbers are uniform across the samples, with no significant differences between Antarctica, Svalbard and Greenland (Table 1). Mean total cell counts in Antarctic cryoconite holes were 5.85 × 10^8^ cells g^–1^, compared with 9.22 × 10^8^ cells g^–1^ in Svalbard and 5.83 × 10^8^ cells g^–1^ in Greenland.

**TABLE 1:** Abundance of microorganisms in cryoconite sediments.

**Location**	**Sample**	**Total cell counts per g of wet sediment**	**95% Confidence Interval**
Svalbard	01	4.85 × 10^8^	0.04
	02	1.82 × 10^9^	0.36
	03	4.61 × 10^8^	0.18
Greenland	04	4.33 × 10^8^	0.20
	05	8.86 × 10^8^	0.22
	06	4.30 × 10^8^	0.02
Antarctica	07	5.27 × 10^8^	0.21
	08	4.62 × 10^8^	0.04
	09	7.68 × 10^8^	0.26

### MPN Counts and Cultivability

**FIGURE 1:**
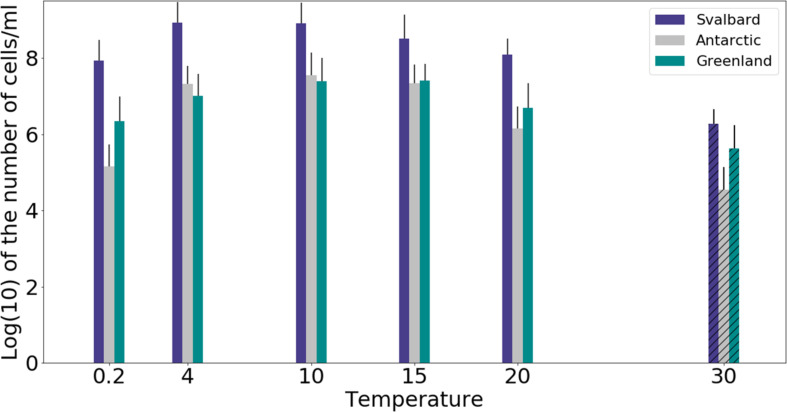
MPN counts of aerobic (oxic) microbial community of cryoconite holes in freshwater medium. Microbial growth was measured by MPN counts as the (average of 3 different cryoconite holes sediments for each location after 71 days of incubation. Samples incubated at 30°C dried out after 30 days, but they are included on the graph for comparison (marked with stripes).

**FIGURE 2:**
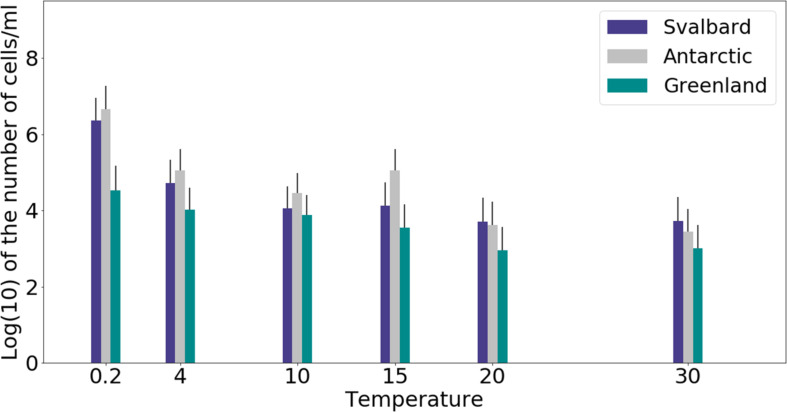
MPN counts of anaerobic (anoxic) microbial community of cryoconite holes in fermenter medium. Microbial growth was measured by MPN counts as the average of 3 different cryoconite holes sediments for each location after 71 days of incubation.

Freshwater medium and fermenter medium inoculated with cryoconite sediment yielded viable cells under oxic and anoxic conditions, and all temperatures tested. Oxic conditions yielded higher numbers of cultivable microorganisms: mean counts of 4.60 × 10^8^g^–1^, 1.30 × 10^7^g^–1^, 1.69 × 10^7^g^–1^ for Svalbard, Greenland, and Antarctic cryoconite respectively (Figure 1), compared with 3.98 × 10^5^g^–1^, 9.61 × 10^3^g^–1^, 8.04 × 10^5^g^–1^ for Svalbard, Greenland, and Antarctic cryoconite in anoxic conditions (Figure 2). The number of cultivable cells in oxic conditions was in the same order of magnitude at each location after 71 days of incubation between 0.2 and 20°C (Figure 1). At 30°C, viable counts were on average 100 times lower than those at 20°C, but these samples unfortunately dried out after 1 month (marked on Figure 1 with stripes). When incubated in an anoxic atmosphere, viability peaked at the coldest temperatures tested (0.2°C) and the number of cultivable cells was comparable between the temperatures from 4 to 30°C (Figure 2).

The cultivability of cryoconite microorganisms was estimated based on total cell counts and MPN counts after 71 days of cultivation. Cryoconite microorganisms yielded very high viable counts under the conditions tested (Table 2). Under oxic conditions, the culturable bacterial count of Svalbard microorganisms was an order of magnitude higher than for Greenland and Antarctic (*p* = 0.00). There were no statistically significant differences between the locations under anoxic conditions. However, it is notable that Antarctic and Svalbard samples have especially high culturability in the lowest temperature tested (0.2°C).

**TABLE 2:** Cultivability of microorganisms from cryoconite holes expressed as% of total cell counts which can be cultured by MPN technique in the aerobic and anaerobic conditions.

	**Anoxic conditions**			**Oxic conditions**		
**Temp (°C)**	**Svalbard**	**Greenland**	**Antarctica**	**Svalbard**	**Greenland**	**Antarctica**
0.2	0.28 ± 0.19	0.01 ± 0.01	0.96 ± 0.79	7.28 ± 0.47	0.48 ± 0.35	0.03 ± 0.01
4	0.01 ± 0.01	0.00 ± 0.00	0.02 ± 0.03	48.38 ± 46.97	1.79 ± 1.25	4.37 ± 3.81
10	0.00 ± 0.00	0.00 ± 0.00	0.01 ± 0.01	53.62 ± 8.79	3.70 ± 2.12	7.65 ± 5.16
15	0.00 ± 0.00	0.00 ± 0.00	0.02 ± 0.03	27.05 ± 2.86	5.98 ± 9.69	5.13 ± 0.07
20	0.00 ± 0.00	0.00 ± 0.00	0.00 ± 0.00	13.16 ± 0.15	1.06 ± 1.46	0.31 ± 0.13
30	0.00 ± 0.00	0.00 ± 0.00	0.00 ± 0.00	0.17* ± 0.11	0.10* ± 0.15	0.01* ± 0.00

*In the oxic conditions at 30°C samples were measured after 23 days.

At all temperatures, the cultivability in anoxic conditions was several orders of magnitude lower than in oxic conditions, with values up to 15200, 3400, and 400 times lower for Svalbard, Greenland, and Antarctica, respectively. However, at 0.2°C, the differences between oxic and anoxic incubations were less pronounced (Table 2).

None of the anoxic samples had statistically significant differences in cultivability. ANOVA, followed by Tukey HSD, revealed that oxic samples from Svalbard and Greenland were statistically different at 0.2, 10, 15, and 20 degrees (*p* = 0.00, *p* = 0.02, *p* = 0.04 and *p* = 0.00, respectively), whereas Svalbard and Antarctic samples were different at 0.2 and 20 degrees (*p* = 0.00 and *p* = 0.00, respectively). There were no statistically significant differences between Greenland and Antarctic samples’ cultivability.

### Microbial Isolates

The highest positive dilution of MPNs was used to inoculate solid agar plates with freshwater medium and isolate the most abundant culturable microbes of cryoconite holes. A total of 44 isolates were isolated and identified by 16S rRNA gene sequencing (Table 3). Svalbard cryoconite samples yielded 13 bacterial isolates and 4 fungi (yeast) isolates, Greenland 12 bacteria and 5 fungi, and Antarctica 10 bacteria, respectively. No yeasts were isolated from Antarctic cryoconite. Most bacterial isolates affiliated with the Actinobacteria (79%), followed by Bacteroidetes (18%) and Proteobacteria (3%). All isolates were capable of fermentation, but did not utilize alternative electron acceptors.

**TABLE 3:** Physiology of microbial isolates of cryoconite holes.

**Isolate**	**Closest relative**	**%**	**Phylum**	**Site**	**Isolation**	**Salinity (%)**	**pH range**	**Substr (%)**	**Freeze-thaw**	**Colony colour**	**T_*max*_ (°C)**
**Bacteria**											
An02O7	Flavobacterium sp. R-36976	99	Bacte	Ant	ox	10	6.5–10	69	1	orange	
An4O7	Flavobacterium sp. R-36976	99	Bacte	Ant	ox	7	6.5–10	66	1	orange	
An15O7	Arthrobacter agilis strain LV7	99	Actino	Ant	ox	6	7–10.5	44	100	yellow	32
An15A7	Tessaracoccus sp. strain AU I5	99	Actino	Ant	anox		7–10.5			yellow	
An15A8	Tessaracoccus sp. strain AU I5	99	Actino	Ant	anox		7–10.5			orange-yellow	
An4A7	Bacterium CS117	99	Actino	Ant	anox	10	4.0–10	74		yellow	36
An4A8	Bacterium CS117	99	Actino	Ant	anox	8	6.5–10.5	38		orange	
An4O8	Marisediminicola sp. N26	99	Actino	Ant	ox	10	6.5–10.5	63	100	orange	
An15O8	Marisediminicola sp. N26	99	Actino	Ant	ox	6.5	6.5–10.5	28	100	orange	
An02O8	Cryobacterium sp. 1021	99	Actino	Ant	ox	8	5–10.5	43	25	red	24
Gr15O6	Frigoribacterium sp. MP117	99	Actino	Gr	ox	8	4–8.5	38	25	orange	
Gr15O5	Frigoribacterium sp. MP117	99	Actino	Gr	ox	2.5	4–8.5	17	25	orange	
Gr02A4	Antarctic bacterium 2CA	99	Actino	Gr	anox	10	4–8.5	28		pale yellow	
Gr15O4	Glaciihabitans tibetensis strain TGC-6	99	Actino	Gr	ox	2	4–8.5	16	25	orange-yellow	
Gr4O4P	Uncultured Bacteroidetes clone IC4058	99	Bacte	Gr	ox				5	pink	
Gr4O6	Rugamonas rubra strain HCR18a	99	Proteo	Gr	ox	0.75	4.5–8	50	25	white	31
Gr4A5	Cryobacterium sp. MDB2-A-1	99	Actino	Gr	anox	10	4–8.5	72		pale yellow	24
Gr02O4	Cryobacterium psychrotolerans MLB-34	99	Actino	Gr	ox	8	4.0–10	59	5	yellow	
Gr02A6	Cryobacterium psychrotolerans ZS14-85	99	Actino	Gr	anox	10	4.0–10	41		pale yellow	
Gr02A5	Cryobacterium sp. MDB1-44	99	Actino	Gr	anox	10	4–9.5	50		pale yellow	
Gr4A4	Cryobacterium sp. MDB1-44	99	Actino	Gr	anox	10	4–8.5	42		pale yellow	
Gr4A6	Cryobacterium sp. MDB1-44	99	Actino	Gr	anox	10	4–8.5	70		pale yellow	
Sv4A3	Cryobacterium sp. MDB1-44	99	Actino	Sv	anox	10	4–8.5	67		pale yellow	
Sv4A2	Cryobacterium sp. MDB2-A-1	99	Actino	Sv	anox	8	4–8.5	48		pale yellow	25
Sv15A2	Cryobacterium sp. MDB2-A-1	99	Actino	Sv	anox	5.5	4–8.5	23		yellow	
Sv02A1	Antarctic bacterium 2CA	99	Actino	Sv	anox	5.5	4–8.5	23		yellow	
Sv4A1	Antarctic bacterium 2CA	99	Actino	Sv	anox	8	4–8.5	66		pale yellow	34
Sv02A3	Antarctic bacterium 2CA	99	Actino	Sv	anox	8	4–8.5	21		yellow	
Sv4O2	Uncultured bacterium clone LE201D02	99	Bacte	Sv	ox	10	6.0–10	7	25	orange	
Sv02O2	Flavobacterium sp. KJF4-15	99	Bacte	Sv	ox	8	6.0–10	47	5	orange	25
Sv02A2	Flavobacterium sp. TMS1-10 16S	99	Bacte	Sv	anox	8	4–9.5	34		yellow	
Sv15A1	Cellulomonas cellasea strain WB102	99	Actino	Sv	anox		2.5–8			pale yellow	
Sv15O1	Frigoribacterium sp. Ha8	99	Actino	Sv	ox	2	5.0–10	54	5	yellow	31
Sv15A3	Actinobacterium Muzt-D93	99	Actino	Sv	anox		4–8.5			pale yellow	
Sv15O3	Glaciihabitans tibetensis strain SD-70	99	Actino	Sv	ox	2	4–8.5	41	25	orange	
**Fungi**											
Gr02O5	Basidiomycota sp. TP-Snow-Y1	91	Basidio	Gr	ox	10	3–8.5	41	100	pale pink	
Gr4O5	Basidiomycota sp. TP-Snow-Y1	91	Basidio	Gr	ox	8	5.5–8.5	27	100	pale pink	19
Gr4O4	Basidiomycota sp. TP-Snow-Y1	91	Basidio	Gr	ox	10	3–8.5	24	100	pale pink	
Gr02O4w	Basidiomycota sp. TP-Snow-Y1	91	Basidio	Gr	ox	10	3–8.5	33		white	18
Gr02O6	Basidiomycota sp. TP-Snow-Y1	91	Basidio	Gr	ox	7	3–8.5	34	100	pale pink	
Sv02O1	Basidiomycota sp. TP-Snow-Y1	92	Basidio	Sv	ox	5	3.0–10	48	100	pale pink	
Sv4O1	Basidiomycota sp. TP-Snow-Y1	92	Basidio	Sv	ox	8	3–8.5	23		pale pink	
Sv02O3	Mrakia sp. isolate J-36	99	Basidio	Sv	ox	10	3.0–10	52	100	white	22
Sv4O3	Mrakia robertii isolate J-127	100	Basidio	Sv	ox	10	3.0–10	66	100	orange	

*Closest relative of isolates is based on rRNA gene sequencing and similarity is expressed in percent (%). Isolation was done in oxic (ox) or anoxic (anox) conditions. Carbon substrates utilization (substr) is expressed as the percent used of tested. “Salinity” is the maximum salinity and “pH range” is the range of media pH in which positive growth was recorded. All the isolates were able to grow at lowest tested salinity of 0.1%. Freeze-thaw survival is the number of cycles after which a culture was viable. Tmax is the maximum temperature that growth could be detected. All tested isolates grew at the lowest tested temp (1°C). Phyla detected include Actinobacteria (Actino), Bacteroidetes (Bacte), and Proteobacteria (Proteo). Sampling sites are Antarctica (Ant), Greenland (Gr), and Svalbard (Sv).*

### pH Tolerance

**FIGURE 3:**
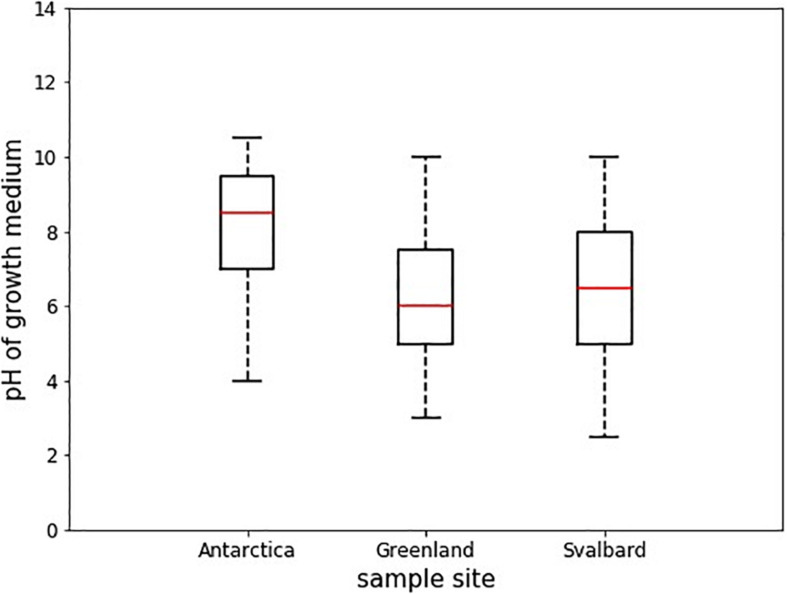
Boxplots showing the median pH tolerance of all microbial isolates from cryoconite holes from Greenland, Svalbard, and Antarctica. The pH of all incubations with positive growth after 30 days (*n* = 43) was noted, and compared between the locations. The red line depicts the median pH, the box envelops an interquartile range and the whiskers mark the 97th centile. There were no outliers.

Antarctic isolates grew in medium with pH ranging from 4 to 10.5, whereas those from Svalbard grew in 2.5 to 10, and those from Greenland, 3 to 10 (Table 3). Comparison of the differences in these pH ranges was analyzed by ANOVA followed by Tukey HSD and showed that the pH range tolerated by Antarctic isolates was significantly different from Svalbard and from Greenland (*p* = 0.00 and *p* = 0.00, respectively) (Figure 3). The mean pH of the successful growth media was highest in the Antarctic samples; the mean pH of media with detectable growth was 8.2, compared to 6.2 for Greenland and 6.5 for Svalbard. There was no statistical difference between oxic and anoxic conditions, or between yeasts and bacteria. Most yeasts grew at pH ≥ 3; Svalbard yeasts were able to grow from pH 3 to 10, although interestingly yeasts from Greenland samples that were assigned to the same OTUs could only grow at pH 3 to 8.5 (Table 3). One bacterial isolate grew at pH 2.5, but the remainder did not tolerate pH ≤ 4.

### Salinity Tolerance

The microbial isolates from cryoconite holes were able to grow in a surprisingly wide salinity range (Table 3). Most (34 out of 39) were able to grow in up to 5% salinity (∼42000 μS cm^–1^). The highest tested salinity of growth medium was 10% (∼77000 μS cm^–1^), where 16 isolates tested positive for growth (Supplementary Figure 1). Interestingly, there was no significant difference of maximum salinity tolerance between yeasts and bacteria from Svalbard and Greenland, nor between isolates from the different sites of origin, or those isolated under anoxic or oxic conditions.

### Temperature Range

Twelve isolates were tested to determine the temperature range in which they were able to grow (Table 3). All tested isolates grew at the lowest tested temp (1°C). All of the bacterial isolates were able to grow above 22°C, with Antarctic isolate AN4A7 having a maximum growth temperature of 36°C, whereas yeasts were limited to 22°C.

### Freeze-Thaw Survival

**FIGURE 4:**
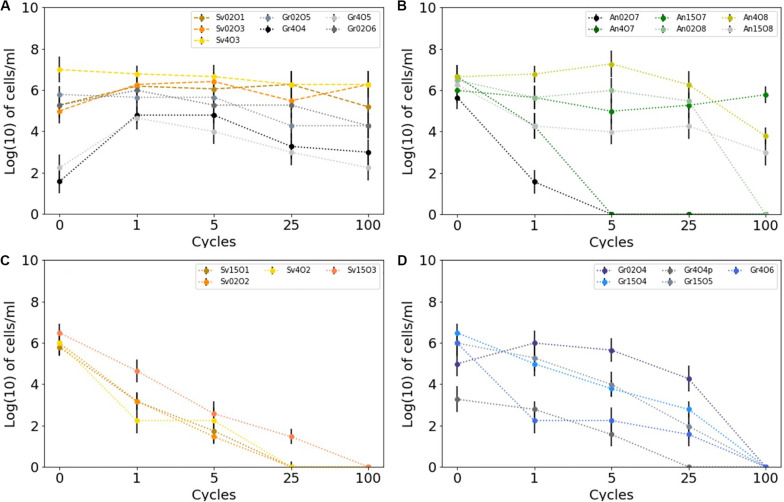
Survival of cryoconite isolates subjected to multiple freeze-thaw cycles as measured by MPN technique. Error bars depicts 95% confidence levels calculated for MPNs. **(A)** Greenland and Svalbard yeasts, **(B)** Antarctic bacteria, **(C)** Svalbard bacteria, **(D)** Greenland bacteria.

Isolates from all sample locations had a mixed response to freezing: some isolates survived multiple freeze-thaw cycles without losing viability (e.g., Antarctic isolate An15O7), whereas others did not (e.g., Antarctic isolate An02O7) (Figure 4). All the yeast strains survived numerous (>100×) freeze and thaw cycles without a significant decrease in viability, when assessed by MPN. In some yeast strains cell counts increased after 100 cycles, whereas others showed a slight decrease, but none were completely inviable (Figure 4A). Two of the yeast isolates (Gr4O5 and Gr4O4) increased in viable cell counts after a single cycle, which might indicate an adaptation to freeze-thaw stress and cells transitioning from non-culturable to culturable state.

Bacterial isolates showed greater variability in response to freeze-thaw stress. The majority (10 out of 15) of the cultures tested belong taxonomically to Gram-positive bacteria (Actinobacteria). Of these, 8 out of 10 remained viable after 25 cycles, and three (all from Antarctica) were viable after 100 cycles. In contrast, none of the Gram-negative strains survived 100 cycles, and they generally lost culturability more rapidly after freeze-thaw stress. The viability of cultures following treatment was variable, regardless of the sampling site. However, the bacteria isolated from Antarctic cryoconite differed from the Arctic (Svalbard, Greenland): the only three bacterial isolates (An15O7, An4O8, An15O8) able to survive 100 cycles of freezing and thawing came from Antarctic samples, although it should also be noted that some Antarctic isolates survived only 1–5 cycles (Figure 4B). Svalbard bacteria viability decayed rapidly, with only one able to survive 25 freeze-thaw cycles (Figure 4C). Greenland bacteria ceased to be viable between 25 and 100 cycles (Figure 4D). Freeze-thaw survival appears to follow phylogeny and hence cellular structure, as isolates belonging to the same genus but obtained from different sites showed a similar response. For example, *Cryobacterium* sp. from Antarctica and from Greenland survived well during the initial cycles and collapsed after 25 cycles. *Flavobacterium* sp. from Svalbard and Antarctica did not cope well and survived 1 to 5 cycles.

There was no significant correlation between the pigmentation of the strains, resistance to salinity and/or extreme pH and freeze-thaw survival (Table 3). Among the isolates with the highest freeze-thaw resistance (25−100 cycles), there were cultures which survived only salinities up to 0.75% (isolate Gr4O6), but also up to 10% (An4O8); dark pigmented isolates (orange) which survived one cycle (A02O7) and 25 cycles (Gr15O6); and strains able to survive extreme pH 10 with both minimal (1 cycle only, An02O7) and good freeze-thaw tolerance (25 cycles, Sv4O2). The three isolates most resistant to freezing (Antarctic isolates An15O8, An4O8, An15O7) were pigmented, resistant to pH 10.5 and able to cope with high salinities (6.5, 10 and 6%, respectively).

### Substrate Test

The cultures tested utilized a wide range of carbon sources from different groups (carboxylic acids, simple and complex carbohydrates, amino acids, alcohols and polyalcohol and other complex substances). There was no strong preference for one single type of carbon source such as carboxylic acids, carbohydrates or amino acids (Table 4), and some isolates were able to live on almost all substrates tested (e.g., genera *Flavobacterium* sp. or *Cryobacterium* sp.). There are some exceptions, for example most isolates belonging to *Antarctic bacterium* species only utilized a small selection of carboxylic acids.

**TABLE 4:** Mean utilization of carbon substrates by cryoconite microorganisms expressed as proportion of all carbon substrates tested in percent.

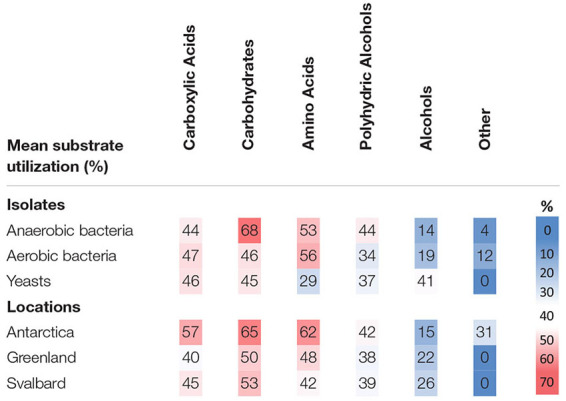

*All the isolates are divided into groups based on isolation or location. Colors indicate a heatmap of substrates’ utilization, with red hues representing a higher proportion of substrates being utilized by selected isolates and blue hues representing low proportion to none substrates being utilized.*

Antarctic isolates used a bigger pool of substrates (45% of those tested) when compared to Svalbard (34%) and Greenland isolates (32%), however, the differences were not statistically significant. Interestingly, Antarctic isolates were the only ones able to utilize the “other” substrates (Supplementary Table 1) which include “non-competitive” choline, betaine and methylamine. Yeasts had a greater capability to use alcohols and were less likely to use amino acids when compared to bacteria. Microbes isolated in anoxic conditions decomposed more types of carbohydrates than those obtained under oxic conditions.

## Discussion

### Microbial Abundance and Growth

The spread of cell numbers reported in the literature is astonishingly wide. After excluding the very extreme values of 0.5−7.5 × 10^14^ cells g^–1^ in Svalbard (Kastovská et al., 2005), the cell numbers reported in cryoconite holes worldwide are between 10^6^ and 10^9^ cells g^–1^ (Stibal et al., 2006; Anesio et al., 2010; Hodson et al., 2010; Singh et al., 2014; Telling et al., 2014; Musilova et al., 2015; Cook et al., 2016) and our data are within this range. Svalbard, Greenland, and Antarctic samples showed no significant differences in the cell counts (mean counts of 9.2, 5.8, and 5.8 × 10^8^ cells g^–1^, respectively).

The microbial diversity and community structure of cryoconite holes is often based on only “snapshots” of the community measured at a given place and time (Edwards et al., 2013), so there is clearly variability in reported results. However, many of the microorganisms isolated in this study have been identified elsewhere (Supplementary Table 2), for example, Flavobacteria class is often dominant in freshwater polar environments (Michaud et al., 2012) and the Cytophaga-Flavobacteria group, to which 18% of isolates in this study belong, was found to be dominant (87.2%) in Canada Glacier cryoconite holes from Antarctica (Foreman et al., 2007). *Flavobacterium, Cryobacterium*, and *Arthrobacter* spp. were also isolated in Antarctic cryoconite holes from Canada Glacier (Christner et al., 2003). *Cryobacterium* spp. was also common in Svalbard soils (Hansen et al., 2007) and basidiomycetous yeasts were predominant in Svalbard sea and glacial ice (Gunde-Cimerman et al., 2003). Lutz et al. (2019) found Cyanobacteria, Proteobacteria, and Actinobacteria phyla to be the most abundant in Antarctic cryoconite holes from Queen Maud Land. The dominance of Actinobacteria, in this study, followed by Bacteroidetes and Proteobacteria, is therefore consistent with these previous findings.

Our experiments showed that oxic conditions yielded higher numbers of viable, cultivable microorganisms than anoxic. Cryoconite holes are largely oxygen rich environments, with aerobic metabolisms and consequent dominance of aerobic microorganisms, but there are anoxic niches (Poniecka et al., 2018) where a thriving anaerobic community can be found. Oxic microorganisms have quite uniform viable counts across the temperatures of 0.2 to 20°C, with visible decline at 30°C, whereas the highest cultivability in anoxic conditions was observed at 0.2°C and the majority of the anaerobic community was still growing at 30°C. It is interesting to speculate the cause of the maximal cultivability at lower temperatures. The anaerobic part of the community could be specifically adapted and active at the times of lower melt. During initial spring melt, there is a lack of mixing by meltwater that can lead to anoxic zones and an ionic pulse (Telling et al., 2014) likely supports higher metabolic activity. Facultative anaerobic heterotrophs may therefore be important in the reactivation of the community after the polar night (Vick-Majors et al., 2014).

### Limits of Cryoconite Microorganisms

Cryoconite microorganisms were able to grow in a wide range of pH values. Reported pH of cryoconite holes in Antarctica was pH ∼6–11 (Porazinska et al., 2004; Tranter et al., 2004; Bagshaw et al., 2007; Stanish et al., 2013), in Svalbard ∼4.7–8.6 (Kastovská et al., 2005; Singh and Singh, 2012), in Greenland ∼4.35 to 6.7 (Chandler, 2012, unpublished; Stibal, unpublished; Black and Bloom Team, 2016, unpublished), and in the Alps ∼5 (Margesin et al., 2002). To some extent, this growth range reflects the physical differences between Arctic and Antarctic holes, with Antarctic microorganisms growing in the highest pH in the laboratory and values up to pH 11 being measured in the field (Tranter et al., 2004). Most of Antarctic cryoconite isolates (7 out of 10) were able to grow in pH 10.5, suggesting that some of them could perhaps withstand even more alkaline conditions. At the other end of the spectrum are the yeast isolates, which seem to be more acidophilic than bacteria, tolerating pH of 3. The individual strains had specific tolerances that were similar to those previously published; for example, *Arthrobacter agilis* strain L77 from a lake in Himalayas (water pH 8.7 to 9.1) had a pH range of 6–9 and tolerated 5% salinity (Singh et al., 2014), whereas *Arthrobacter agilis* strain LV7 in this study from Antarctic cryoconite hole had a pH range of 7–10.5 and tolerated 6% salinity.

Tolerance of high salinity was a universal trait regardless of the sampling site, with all the isolated microorganisms able to grow outside the salinities typically found in cryoconite holes and on the glacier surfaces. Electrical conductivity (EC) of cryoconite holes in southwest Greenland was 2.2–3 μS cm^–1^ (Chandler, 2012, unpublished) and in Antarctica 5–20 μS cm^–1^ on Canada Glacier (Bagshaw et al., 2011) and ∼60–110 μS cm^–1^ on Taylor Glacier (Porazinska et al., 2004). This compares with adjacent habitats which frequently have extreme EC: Lake Hoare and Lake Bonney (Taylor Valley) were 65–7798 μS cm^–1^ (Courtright et al., 2001); Fresh, Orange, and Salt Ponds on the McMurdo Ice Shelf were 158, 937, and 52900 μS cm^–1^ respectively (Jungblut et al., 2005) and soils in Wright Valley > 1500 μS cm^–1^ (Courtright et al., 2001). In Svalbard, in the forefield of Midtre Lovenbreen, a 2347-year-old permafrost soil was ∼8200 μS cm^–1^ (Hansen et al., 2007). These habitats are likely important inoculum for cryoconite holes (Porazinska et al., 2004; Bagshaw et al., 2013). Salinity tolerance may also assist in freeze-thaw protection: cryoconite holes undergo multiple freeze-thaw events in their lifetime, so microorganisms must either survive freezing, or avoid it by persisting in high salinity brine veins within the ice crystal structure (Mader et al., 2006; Telling et al., 2014). Fungi isolated from Svalbard sea ice and glacial ice grew better on halotolerant media than on the traditional media, with fungal growth up to 24% salinity, indicating that a high number of halophilic species can be found on glaciers and sea ice (Gunde-Cimerman et al., 2003). Another possibility is that resistance to salinity is the by-product of resilience to other environmental conditions such as high UV, dehydration or freezing (Poli et al., 2010). Cyanobacteria from hypersaline ponds on McMurdo Ice Shelf use organic osmolytes as a protection from osmotic stress (Jungblut et al., 2005) and large quantities of EPS were found in the brine channels in sea ice (Nichols et al., 2005). Mechanical damage to cell walls during freezing results either from intracellular ice crystals formation or recrystallization of extracellular small ice crystals into large grains, or by osmotic stress caused by dehydration following extracellular freezing and electrolyte concentration in the remaining liquid phase (Fonseca et al., 2001; Pegg, 2007; Raymond, 2016). The similar mechanisms of cell damage by dehydration, freezing and hypersaline solution often results in cross-protection, however, there was no correlation between high salinity resistance and other variables such as freeze-thaw survival in this study.

Physical damage sustained to the cell during freezing depends on its shape, structure and membrane rigidity (Jordan et al., 2008; Mai-Prochnow et al., 2016), hence it might explain some of the differences between the isolates. Yeasts have a thick cell wall composed of polysaccharides. Gram-positive bacteria have a thick (20–80 nm), rigid cell wall built of peptidoglycan, and Gram-negative bacteria have a thin wall. Gram-positive bacteria were classically considered to be typical for soil ecosystem and consequently adapted to dry conditions (Barka et al., 2016). In this study, all the yeasts survived well, and typically Gram-positive bacteria generally survived better (almost all survived 25 cycles and some survived 100) than Gram-negative (none survived 100 cycles). The structure of cell envelope has a major impact, but additional protection against concomitant damaging factors such as reactive oxygen species generated during thawing can be achieved by carotenoids – pigments which protect from photosensitization and from reactive oxygen species (Dahl et al., 1989; Park et al., 1997; Dieser et al., 2010; Mai-Prochnow et al., 2016). Most of the isolated cryoconite hole microorganisms are pigmented (Table 3). Another protective strategy is excreting protective antifreeze proteins and/or EPS (Poli et al., 2010; Sathiyanarayanan et al., 2015; Raymond, 2016; Perkins et al., 2017). Many cryophilic genera found also in cryoconite holes were shown to produce EPS, such as *Flavobacterium* sp. (Nichols et al., 2005; Sathiyanarayanan et al., 2015) or *Arthrobacter* sp. (Singh et al., 2014). The *Arthrobacter* genus was reported to survive multiple freeze thaw cycles (Muñoz et al., 2017). Moreover, numerous cryosphere bacteria show antifreeze proteins activity, including Actinobacteria and Bacteroidetes from Antarctic moss (Raymond, 2016), *Sphingomonas, Plantibacter, Pseudomonas*, and *Arthrobacter* sp. from Antarctic ice and sediments (Muñoz et al., 2017), or *Cryobacterium, Pseudomonas*, and *Subtercola* sp. among Arctic cryoconite bacteria (Singh et al., 2014). Most of the isolated bacteria in this study belong to Actinobacteria, and there are also several isolates of *Cryobacterium* from all locations.

Several isolates were tested to estimate the temperature range in which they were able to grow. Bacterial isolates were psychrotolerant, all being able to grow above the arbitrary threshold for psychrophilic growth of 22°C (Cavicchioli, 2016). Yeasts, which only grew in the lower incubation temperatures and were able to better withstand freezing-thawing cycles, were psychrophiles. Although the sample size is small, it is interesting to debate the difference between bacteria and yeasts isolated form these cold environments. The results suggest that yeasts are better adapted to lower temperatures and stresses encountered in glacial environments such as freeze-thaw. Conversely, bacteria present a greater variability in their physiology, with some species adapted as well as yeasts to freeze-thaw and with similarly good salinity tolerance and broad pH range.

Survival of bacteria is therefore determined by multiple factors, including the structure of the cell envelope, internal pigments, excreted antifreeze proteins and compatible solutes. As the detailed mechanisms by which each species survives freeze-thawing were not the aim of this study, we can only speculate the cause of the differences. It was notable that yeasts are very resistant to freezing, yet they are not found as the dominant group in other studies of cryoconite community. The most abundant retrievable microorganisms of cryoconite holes are resistant to a wide range of fluctuating environmental conditions and stressors. Such conditions, including freeze-thaw cycles, high salinities, temporary anaerobic conditions and pH variability are typically encountered in arid polar soil habitats (Courtright et al., 2001; Paul, 2006; White, 2006; Poage et al., 2008; Bagshaw et al., 2013), which are likely to be seeding grounds for cryoconite holes.

### Organic Carbon Utilization and Metabolic Capabilities

It is well known that organic matter is plentiful in cryoconite holes (Tranter et al., 2004; Musilova et al., 2016) and that there are genes present for biodegradation (Edwards et al., 2013) that are able to decompose organic matter (Sanyal et al., 2018). Thus, unsurprisingly, we detected strains which are known to decompose organic matter [e.g., *Flavobacterium* sp. (Williams et al., 2013), *Cryobacterium* sp. (Sanyal et al., 2018)]. Overall, cryoconite holes microorganisms produce a variety of enzymes for different groups of carbon substrates, suggesting they are effective in scavenging carbon substrates when and if they become available. Isolates obtained under anoxic conditions tend to utilize a higher proportion of carbohydrates when compared to those obtained aerobically. Facultatively anaerobic microorganisms have a different type of metabolism, depending on the availability of oxygen, and will commonly use monomeric sugars as their electron acceptor, hence it is unsurprising that they specialize in carbohydrates. Antarctic bacteria have the capacity to use a bigger pool of substrates, including “non-competitive” organic matter such as choline and betaine, when compared to the Arctic microorganisms. This can be explained by a smaller input of carbon sources due to entombment of Antarctic cryoconite holes (Telling et al., 2014; Webster-Brown et al., 2015), whereas the Arctic holes are frequently flushed with melt water during the melt season. Whilst the differences are not statistically significant, due to a very high variability among isolates, none of the Arctic isolates utilize “non-competitive” carbon substrates.

Some closely related isolates assigned to the same OTU showed striking differences in their metabolic capabilities. Without further studies it is impossible to pinpoint the source of the differences, but possible reasons include the initial incubation temperature, fitness of the culture and the metabolic impact of genome rearrangement in stressed and starving microorganisms. Such differences are quite common, for example *Cellulomonas* strains can have differences in their physiology (Hatayama et al., 2013), even when they are closely related (∼98% sequence similarity, which is enough to assign them to the same OTU). Another example is *Flavobacterium* genus, which is also widespread in cryoconite holes, where two distinct species had 98.9% 16S rRNA gene sequence similarity (Peeters and Willems, 2011). The resolution of 16S rRNA gene at species level is often limited, as it is a highly conserved gene (Peeters and Willems, 2011). Isolates which share the same OTU are not necessarily the same bacterium, it only means that they have a high degree of similarity in one gene. Even if they have highly similar 16S rRNA genes, their entire genome is not likely to be the same (Woese, 1987; Rosselló-Mora and Amann, 2001). Finally, culturing is a selective process, so it is possible that a single bacterium which gave a pure isolate was devoid of an enzymatic pathway. Therefore, while it is useful to investigate the capabilities of the organisms, we cannot exclude that in the environment their metabolic capabilities could differ, depending on overall fitness and competition. Regardless of these differences, microorganisms of cryoconite holes clearly complement each other and partly specialize in the types of substrates used. We might only speculate that it depends on the microniches within the cryoconite holes and the relationships with other microorganisms, as well as the availability of particular substrates, but there is not enough data in the literature on the composition of cryoconite organic matter to draw clearer conclusions. What can be concluded from this experiment is that isolates in pure culture were able to utilize a broad range of carbon substrates and as a community, they can scavenge almost all substrates tested. They seem well adapted to the extremely low organic matter content, which is typically encountered in glacial environments (Anesio and Laybourn-Parry, 2012) and barren Antarctic soils (Poage et al., 2008).

## Conclusion

Microorganisms inhabiting cryoconite holes are exposed to a wide range of extreme conditions, and this study demonstrates their broad tolerance. The generally oxygen-rich cryoconite holes harbor an active, culturable anaerobic community. Anaerobic cultivability is better in the coldest conditions tested, which suggests their adaptation to and dominance of the beginning or end of the melt season, when anoxic conditions are likely to occur. Apart from anoxia, cultured microorganisms can withstand a wide range of other physical stresses, including extreme pH and salinity. As pH tolerance broadly reflected the values found in the samples locations, this may indicate that extreme salinities could also be found in cryoconite holes in certain conditions, for example, during freeze-thaw cycles, or that the microbial community is seeded from nearby saline habitats, such as arid soils or melt ponds. Cryoconite microorganisms use a wide range of substrates and as a community are effective in scavenging limited carbon sources in cryoconite holes. Their metabolic capabilities seem to depend not only on the genetic affiliation, but also, perhaps, fitness of the culture and phenotypic differences between closely related species. Such phenotypic differences are especially likely, as the bacteria with a high 16S rRNA gene similarity show differences in their physiology. Antarctic isolates showed greater resistance to freezing and thawing cycles and greater use of variable carbon sources when compared to Arctic ones, suggesting they might be adapted to harsher conditions of Antarctic cryoconite holes. However, we found some of the same genera in both Arctic and Antarctic cryoconite holes, similar total cell numbers and the same range of salinities withstood, demonstrating that cryoconite hole microorganisms from both poles also share some physiological traits.

## Data Availability Statement

The datasets generated for this study can be found in the Genbank MT430950, MT432272–MT432304, MT473233, and MT473713–MT473721.

## Author Contributions

EP, EB, HS, MT, and AA contributed to the conception and design of the study. EP, HS, GW, CW, and AS performed the research. EP, EB, and HS analyzed the data. All authors contributed to the manuscript.

## Conflict of Interest

The authors declare that the research was conducted in the absence of any commercial or financial relationships that could be construed as a potential conflict of interest.

## Footnotes

1 blast.ncbi.nlm.nih.gov
