# Supplementary material for: Physiological Capabilities of Cryoconite Hole Microorganisms
Source: Front Microbiol. 2020 Jul 31;11:1783. doi: 10.3389/fmicb.2020.01783 (PMC7412143; doi:10.3389/fmicb.2020.01783)
Supplement: Supplementary file 1 [file Table_1.DOCX]

Supplementary Material

# Supplementary Data

Supplementary table 1. **List of carbon substrates**. Microbial isolates from cryoconite holes were tested for growth in a liquid media with addition of a single carbon source in the laboratory conditions.

| **Substrate** | **Concentration** | **Substrate** | **Concentration** |
| --- | --- | --- | --- |
| *Carboxylic acids*  Formate  Acetate  Propionate  Butyrate  Hexonoate  Malonate  Succinate  Fumarate  Malate  Pyruvate  Lactate  Glycolate  Salicylate  DPA  Glyoxylate  Ketoglutarate  Citrate  *Carbohydrates*  Fructose  Cellulose  Starch  Sucrose  Maltose  Cellobiose  Trehalose  Mannose  Galactose  Xylose  Arabinose  Rhamnose  Glucose  Gluconate  Glucosamine | 20 mM  15 mM  10 mM  10 mM  5 mM  15 mM  15 mM  15 mM  15 mM  15 mM  15 mM  20 mM  2.5 mM  5 mM  20 mM  10 mM  10 mM  5mM  0.1%  1%  1 mM  1 mM  0.2 mM  0.5 mM  1 mM  1 mM  1 mM  1 mM  1 mM  5 mM  5 mM  5 mM | *Amino acids*  Peptone  Casamino Acids  Yeast extract  Serine  Alanine  Arginine  Glutamate  Proline  Isoleucine  Lysine  Cysteine  Methionine  Phenylalanine  *Polyhydric alcohols*  Sorbitol  Mannitol  Inositol  *Alcohols*  Erythritol  Glycerol  Glycol  Methanol  Ethanol  Butanol  Ethanolamine  *Other*  Choline  Betaine  Methylamine | 0.25%  0.25%  0.5%  15 mM  10 mM  10 mM  10 mM  10 mM  6.25 mM  5 mM  10 mM  10 mM  5 mM  1 mM  5 mM  5 mM  10 mM  10 mM  10 mM  10 mM  10 mM  5 mM  15 mM  15 mM  15 mM  20 mM |


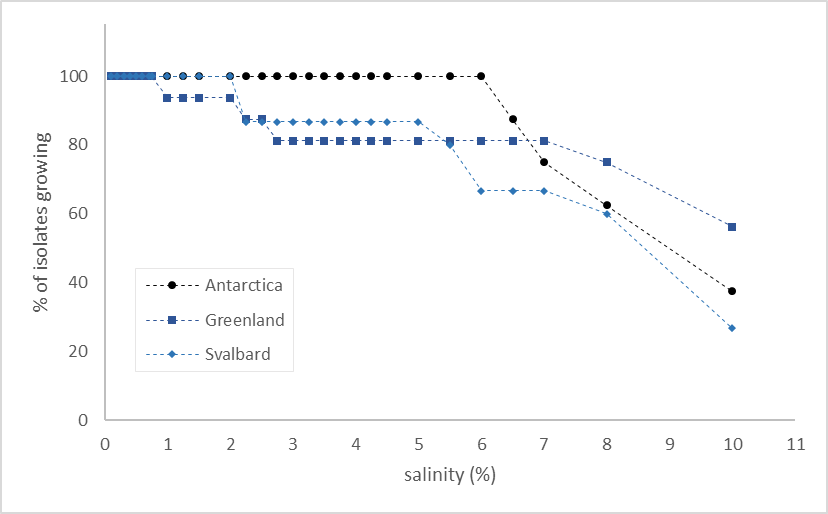


Supplementary Figure 1. Percentage of total number of microbial isolates tested growing in the medium with increasing salinity.

**Supplementary table 2.** Closest relative of microbial isolates from cryoconite holes. Closest relative is based on rRNA gene sequencing and similarity is expressed in percent (%). Isolation source and location of closest relative is sourced from Genbank database.

| **Isolate** | **Site** | **Closest relative** | **%** | **Closest relative source** | **Closest relative location** |
| --- | --- | --- | --- | --- | --- |
| Bacteria |  |  |  |  |  |
| An02O7 | Ant | Flavobacterium sp. R-36976 | 99 | aquatic microbial mat | Antarctica |
| An4O7 | Ant | Flavobacterium sp. R-36976 | 99 | aquatic microbial mat | Antarctica |
| An15O7 | Ant | Arthrobacter agilis strain LV7 | 99 | lake cyanobacterial mat | Antarctica |
| An15A7 | Ant | Tessaracoccus sp. strain AU I5 | 99 | marine macroalgae | Antarctica |
| An15A8 | Ant | Tessaracoccus sp. strain AU I5 | 99 | marine macroalgae | Antarctica |
| An4A7 | Ant | Bacterium CS117 | 99 | cryoconite hole | Antarctica |
| An4A8 | Ant | Bacterium CS117 | 99 | cryoconite hole | Antarctica |
| An4O8 | Ant | Marisediminicola sp. N26 | 99 | permafrost | Antarctica |
| An15O8 | Ant | Marisediminicola sp. N26 | 99 | permafrost | Antarctica |
| An02O8 | Ant | Cryobacterium sp. 1021 | 99 | Glacier No.1 | China |
|  |  |  |  |  |  |
| Gr15O6 | Gr | Frigoribacterium sp. MP117 | 99 | ice water (glacier) | Tibet |
| Gr15O5 | Gr | Frigoribacterium sp. MP117 | 99 | ice water (glacier) | Tibet |
| Gr02A4 | Gr | Antarctic bacterium 2CA | 99 | glacier sediment | Antarctica |
| Gr15O4 | Gr | Glaciihabitans tibetensis strain TGC-6 | 99 | cryoconite hole | Greenland |
| Gr4O4P | Gr | Uncultured Bacteroidetes clone IC4058 | 99 | ice core | Svalbard |
| Gr4O6 | Gr | Rugamonas rubra strain HCR18a | 99 | cryoconite hole | Himalaya |
| Gr4A5 | Gr | Cryobacterium sp. MDB2-A-1 | 99 | glacier | China |
| Gr02O4 | Gr | Cryobacterium psychrotolerans strain MLB-34 | 99 | cryoconite hole | Svalbard |
| Gr02A6 | Gr | Cryobacterium psychrotolerans strain ZS14-85 | 99 | soil | Antarctica |
| Gr02A5 | Gr | Cryobacterium sp. MDB1-44 | 99 | glacier | China |
| Gr4A4 | Gr | Cryobacterium sp. MDB1-44 | 99 | glacier | China |
| Gr4A6 | Gr | Cryobacterium sp. MDB1-44 | 99 | glacier | China |
|  |  |  |  |  |  |
| Sv4A3 | Sv | Cryobacterium sp. MDB1-44 | 99 | glacier | China |
| Sv4A2 | Sv | Cryobacterium sp. MDB2-A-1 | 99 | glacier | China |
| Sv15A2 | Sv | Cryobacterium sp. MDB2-A-1 | 99 | glacier | China |
| Sv02A1 | Sv | Antarctic bacterium 2CA | 99 | glacier sediment | Antarctica |
| Sv4A1 | Sv | Antarctic bacterium 2CA | 99 | glacier sediment | Antarctica |
| Sv02A3 | Sv | Antarctic bacterium 2CA | 99 | glacier sediment | Antarctica |
| Sv4O2 | Sv | Uncultured bacterium clone LE201D02 | 99 | arctic river | Russia |
| Sv02O2 | Sv | Flavobacterium sp. KJF4-15 | 99 | subarctic fjord | Svalbard |
| Sv02A2 | Sv | Flavobacterium sp. TMS1-10 16S | 99 | glacier | China |
| Sv15A1 | Sv | Cellulomonas cellasea strain WB102 | 99 | woodchip bioreactor | USA |
| Sv15O1 | Sv | Frigoribacterium sp. Ha8 | 99 | glacier | China |
| Sv15A3 | Sv | Actinobacterium Muzt-D93 | 99 | glacial ice core | China |
| Sv15O3 | Sv | Glaciihabitans tibetensis strain SD-70 | 99 | cryoconite hole | Himalaya |
|  |  |  |  |  |  |
| Fungi |  |  |  |  |  |
| Gr02O5 | Gr | Basidiomycota sp. TP-Snow-Y1 | 91 | glacier surface snow | China |
| Gr4O5 | Gr | Basidiomycota sp. TP-Snow-Y1 | 91 | glacier surface snow | China |
| Gr4O4 | Gr | Basidiomycota sp. TP-Snow-Y1 | 91 | glacier surface snow | China |
| Gr02O4w | Gr | Basidiomycota sp. TP-Snow-Y1 | 91 | glacier surface snow | China |
| Gr02O6 | Gr | Basidiomycota sp. TP-Snow-Y1 | 91 | glacier surface snow | China |
|  |  |  |  |  |  |
| Sv02O1 | Sv | Basidiomycota sp. TP-Snow-Y1 | 92 | glacier surface snow | China |
| Sv4O1 | Sv | Basidiomycota sp. TP-Snow-Y1 | 92 | glacier surface snow | China |
| Sv02O3 | Sv | Mrakia sp. isolate J-36 | 99 | Russell glacier | Greenland |
| Sv4O3 | Sv | Mrakia robertii isolate J-127 | 100 | Russell glacier | Greenland |
|  |  |  |  |  |  |
